# The Effect of Mesenchymal Stem Cell-Derived Extracellular Vesicles on Hematopoietic Stem Cells Fate

**DOI:** 10.15171/apb.2017.065

**Published:** 2017-12-31

**Authors:** Hamze Timari, Karim Shamsasenjan, Aliakbar Movassaghpour, Parvin Akbarzadehlaleh, Davod Pashoutan Sarvar, Sara Aqmasheh

**Affiliations:** ^1^Stem Cell Research Centre, Tabriz University of Medical Sciences, Tabriz, Iran.; ^2^Hematology Oncology Research Centre, Tabriz University of Medical Sciences, Tabriz, Iran.; ^3^Department of Pharmaceutical Biotechnology, Faculty of Pharmacy, Tabriz University of Medical Sciences, Tabriz, Iran.

**Keywords:** Expansion, Graft versus host disease, Hematopoietic stem cells, Mesenchymal stem cell-derived extracellular vesicl, Transplantation

## Abstract

Hematopoietic stem cells (HSCs) are multipotent stem cells, with self-renewal ability as well as ability to generate all blood cells. Mesenchymal stem cells (MSCs) are multipotent stem cells, with self-renewal ability, and capable of differentiating into a variety of cell types. MSCs have supporting effects on hematopoiesis; through direct intercellular communications as well as secreting cytokines, chemokines, and extracellular vesicles (EVs). Recent investigations demonstrated that some biological functions and effects of MSCs are mediated by their EVs. MSC-EVs are the cell membrane and endosomal membrane compartments, which are important mediators in the intercellular communications. MSC-EVs contain some of the molecules such as proteins, mRNA, siRNA, and miRNA from their parental cells. MSC-EVs are able to inhibit tumor, repair damaged tissue, and modulate immune system responses. MSC-EVs compared to their parental cells, may have the specific safety advantages such as the lower potential to trigger immune system responses and limited side effects. Recently some studies demonstrated the effect of MSC-EVs on the expansion, differentiation, and clinical applications of HSCs such as improvement of hematopoietic stem cell transplantation (HSCT) and inhibition of graft versus host disease (GVHD). HSCT may be the only therapeutic choice for patients who suffer from malignant and non-malignant hematological disorders. However, there are several severe side eﬀects such GVHD that restricts the successfulness of HSCT. In this review, we will discuss the most important effects of MSCs and MSC-EVs on the improvement of HSCT, inhibition and treatment of GVHD, as well as, on the expansion of HSCs.

## Introduction

Bone marrow (BM) microenvironment or BM niche plays a significant role in the control of hematopoietic stem cells (HSCs) fate through mesenchymal stem cells (MSCs) and other stromal cells.^1^ HSCs include a very small portion of the BM cells which are multipotent cells, with the self-renewal ability and capable of generating all blood cells.^2,3^ MSCs are multipotent and non-hematopoietic stem cells with self-renewal ability and capability of proliferating and differentiating into several cell types such as adipocytes, osteocytes, chondrocytes, fibroblasts, cartilage, bone, cardiomyocytes, skeletal myocytes, and stromal cells.^4-6^

MSCs are particularly low immunogenic, and due to their immunomodulatory features that affect a broad range of innate and adaptive immune system responses, act as a therapeutic agent in the regenerative medicine to repair injured tissues, tumor inhibition, and immunosuppression.^7,8^ These effects of MSCs in the result of the MSCs differentiation into various cell types, including both mesenchymal and non-mesenchymal cell types and MSCs paracrine molecules, including cytokines, chemokines, growth factors, and extracellular vesicles (EVs).^9,10^ MSCs have supporting function in hematopoiesis in the BM microenvironment through expression of multiple adhesion molecules that are necessary for cell-cell, cell-matrix interactions, homing and mobilization of HSCs, as well as, production of cytokines, chemokines, growth factors, and EVs that affect the HSCs expansion, differentiation, and transplantation.^11-14^

Nowadays, gathering data from investigations show that the most therapeutic effects of MSCs as a result of their paracrine activities.^15,16^ On the other hand, recent studies demonstrated that mesenchymal stem cell-derived extracellular vesicles (MSC-EVs) used as a possible therapeutic agent in many disorders and their biological functions and effects is almost like MSCs.^5,17,18^ EVs from different cell sources including MSCs have been involved in many pathological and physiological processes that also most of them performed by their parental cells such as cell proliferation, cell differentiation, cleaning undesired proteins, genetic exchanges, antigen presentation, angiogenesis, immune system responses, tumor metastasis and inhibition, inflammation, and distributing of oncogenes and pathogens.^19,20^ MSC-EVs are safer than their parental cells due to their specific safety advantages such as the lack of risk for aneuploidy due to their not self-renewal ability, the lower potential to trigger immune system responses and subsequently lower immune rejection due to their very small size (nm) and lower expression of cell surface molecules such as MHC molecules, their preserved function during storage and their preserved cargo versus *in vivo* degradation due to their encapsulated cargo, and limited side effects or toxicity.^21-23^ Moreover, recent *ex vivo* and *in vivo* investigations showed that MSC-EVs therapy can use in the scope of improving hematopoietic stem cells transplantation (HSCT), and HSCs expansion, as well as, treatment of graft versus host disease (GVHD).^12,24,25^

The goals of this article, are to review the most important effects of MSCs and MSC-EVs on the improvement of clinical applications in the scope of HSCT, treatment and inhibition of GVHD following HSCT, as well as, improvement of *ex vivo* expansion of HSCs.

### Characteristics and therapeutic applications of MSC-EVs

EVs are cell-derived vesicles which secreted by a variety of cell types such as MSCs, cytotoxic T cells, mast cells, neurons and other cells into the extracellular milieu.^17,26^‏ EVs include exosomes, microvesicles (also called microparticles or ectosomes), and apoptotic bodies, which are different in size and mechanism of formation.^5,26^ Exosomes are derived from the internal budding of the late endosomes that led to the formation of multivesicular bodies (MVBs) and are released from cells when MVBs fuse with the cell membrane, with the size range from 40 to 100 nm in diameter.^5,17^ Microvesicles (MVs) are derived from the direct outward budding of the cell membrane, with the size range from 50 to 1000 nm in diameter.^5^ Apoptotic bodies are cell fragmentations that released from cells that undergoing apoptosis and are identified via expression of phosphatidylserine on their surface, with the size range from 50 to 5000 nm in diameter.^26^ MSC-EVs express cell surface molecules from their parental cells such as CD29, CD44, CD73, and CD105, as well as, express endosome-associated surface molecules such as CD81, CD82, CD63, CD53, CD9, and CD37. They contain endosome-associated proteins such as TSG101 (tumor susceptibility gene 101), Alix, Flotillin, Annexins, SNAREs, and Rab GTPase, and lipids such as cholesterol, ceramides, and phospholipids, as well as, several types of RNA such as siRNA, miRNA, mRNA and tRNA fragments.^26-28^

EVs have been separated from various biological body fluids such as serum, milk, urine, amniotic fluid, saliva, synovial fluid, and as well as from the supernatant of many cell cultures such as MSCs, dendritic cells, platelets, T cells, B cells, and other cells.^5,17^‏ EVs due to their very small size (nm) could easily be transported through interstitial space, blood and other biological body fluids, even the blood-brain barrier.^29^ Therefore, they exert their effects in the intercellular communications on the target cells via an endocrine effect on distant cells and paracrine effect on adjacent cells.^29^ EVs could be uptake by target cells through direct fusion with the cell membrane and the variety of molecular endocytic pathways such as clathrin-dependent endocytosis, caveolin-dependent endocytosis, phagocytosis, macropinocytosis, and lipid raft-dependent endocytosis. EVs uptake mechanisms depend on types of proteins, glycoproteins, and proteoglycans that located on the membrane of EVs and target cells.^29,30^

MSC-EVs are important mediators in the intercellular communications that change the wide spectrum of pathological and physiological processes of the target cells by transferring of biological molecules from MSCs.^31^ Factors such as inflammatory stimuli, hypoxic conditions, stress, acidic PH, and high levels of intracellular calcium influence the secretion of EVs from MSCs both in pathological and physiological conditions.^32-34^ Recent research activities on the MSC-EVs have shown supporting therapeutic effects in the field of cardiovascular disease, neurological diseases, liver disease, kidney disease, lung disease, immune system disease, cutaneous wound healing, and tumor inhibition.^5,35^ The effect of MSC-EVs in the recent studies on various conditions is summarized in Table 1.

Nowadays, attention has been focused on the production of EVs by genetic engineering from their parental cells for therapeutic goals in order to by engineering EVs that contain therapeutic cargo for targeting particular tissues.^36,37^ For example, exosomes derived from MSCs could decrease renal fibrosis and transforming growth factor β1 (TGF-β1) stimulated damage via delivering exogenous miRNA let7c to damaged kidney cells in the mouse model of unilateral ureteral obstruction.^38^ Also, use of MSC-EVs for active drug delivery that first time reported by Pascucci et al. for inhibiting of pancreatic tumor.^39^ In this regard, MSC-EVs that express surface TRAIL (TNF-related apoptosis-inducing ligand) that is an anti-cancer soluble recombinant protein, could induce apoptosis in 11 TRAIL-resistant cancer cell lines in a dose-dependent way but could not induce apoptosis in primary human bronchial epithelial cells because of TRAIL neutralization or caspase activity inhibition.^40^

### The effect of MSCs on HSCs expansion

The main sources for isolation of HSPCs, including umbilical cord blood (UCB), mobilized peripheral blood with granulocyte colony-stimulating factor (G-CSF), and BM.^48,49^ UCB has important advantages such as easy to acquire, easy availability, less stringent for HLA matching, lower incidence of GVHD, and lower risk for transmission of infectious diseases than peripheral blood and BM. The major disadvantage of UCB is the low number of HSPCs in a cord blood unit, leading to delays in platelets and neutrophils recovery in the peripheral blood after HSCT.^50,51^ To overcome this disadvantage, there are two main ways for increasing the number of HSCs: co-infusion of two cord blood units and *ex vivo* expansion of HSCs.^52^ Co-infusion of two cord blood units has shown some possible improvement than the infusion of single cord blood unit, but hematopoietic recovery still remains sub-optimal with moderate improvement in platelets and neutrophils recovery after HSCT ^53^ and may be increased the incidence of GVHD.^54^ Therefore recent works that have been done, further focusing on the improvement of *ex vivo* expansion methods for increasing the number of HSCs.^55,56^ In numerous protocols, cytokines such as stem cell factor (SCF), thrombopoietin (TPO), and FMS-like tyrosine kinase 3 ligand (FLT3-L) that important for maintaining of HSCs in a more primitive fraction (CD34^+^/CD38-) have been used to promote *ex vivo* expansion of HSCs, and the addition of other cytokines such as interleukin-3 (IL-3), IL-6, IL-11, and G-CSF had optimized HSCs *ex vivo* expansion rate. Nevertheless, most of these protocols confirmed that cytokine-mediated *ex vivo* expansion, shown moderate increases in the number of HSCs with lesser improvement in platelets and neutrophils recovery after HSCT, as well as, promoting differentiation of HSCs to the mature cell lines. For this reasons, the use of MSCs has been suggested for improving *ex vivo* expansion of HSCs in the recent years.^55,57-59^

**Table 1:** Effects of MSCs-EVs on the various conditions

**Source of EVs**	**Type of EVs and their size**	**Isolation method**	**Identify method**	**Administration way of EVs**	**Outcome**	**Ref**
Human UCB-MSCs	Exosome 40-100 nm	Ultracentrifugation (100000 g 1 h)	TEM and western blotting	Direct injection into lobes of mouse liver	Reduce mouse liver fibrosis and hepatic inflammation	^41^
Human fetal tissue MSCs	Exosome Size not shown	HPLC	Not shown	Intrasplenic injection	Improve mouse liver regeneration through increasing of hepatocyte proliferation and survival	^42^
Mouse BM-MSCs	MVs 80-1000 nm	Ultracentrifugation (100000 g 1 + 1 h)	Flowcytometry, TEM, and SEM	Intravenous injection (tail)	Promote renal function and survival in NOD/SCID mice with AKI	^43^
Human UCB-MSCs	MVs 20-1000 nm	Ultracentrifugation (100000 g 1 + 1 h)	NTA, TEM, and SEM	Co-culture (*ex vivo*) Injection into left carotid artery of rat	MVs with or without IFN-γ stimulation suppress T-cell proliferation through increasing the percentage of CD4^+^CD25^+^FOXP3^+^ Treg cells *ex vivo*. Only MVs without IFN-γ stimulation preserved rats kidney from AKI *in vivo*	^32^
Human WJ-MSCs	MVs 30-500 nm	Ultracentrifugation (100000 g 1 + 1 h)	TEM and flowcytometry	Co-culture (ex vivo) Intravenous injection	*Ex vivo* suppress the expression of CX3CL1 (chemotactic factor for macrophages) in HUVECs under hypoxia-induced damage. Improve ischemia/reperfusion AKI in rats by increased proliferation, and decreased inflammation and apoptosis of renal cells *in vivo*	^44^
Human ESC-MSCs	Exosome 50-100 nm	Ultrafiltration and HPLC	Electron microscopy	Intravenous injection (tail)	Decrease myocardial ischemia/reperfusion damage in a mouse model through reduction of infarct size	^22^
Mouse BM-MSCs and Human WJ-MSCs	Exosome 30-100 nm	Ultrafiltration and size-exclusion chromatography	Electron microscopy	Intravenous injection (left jugular and tail vein)	Suppress hypoxic pulmonary hypertension in a murine model by inhibition of hypoxia-activated signaling pathway that causes lung inflammation	^34^
Rat BM-MSCs	Exosome Peak 116 ± 49 nm by qNano system	ExoQuick-TC^TM^ kit	qNano system, TEM, western blotting, and confocal fluorescence microscopy	Intravenous injection (tail)	Promote functional recovery through increased endogenous brain angiogenesis and neurogenesis, as well as, decreased neuroinflammation in a rat model of TBI	^45^
	40-120 nm by TEM					
Human MSCs	EVs Mean 100 nm	Anion exchange chromatography	Not shown	Intravenous injection (tail)	Efficiently suppress autoimmunity in murine models of IDDM and EAU. Suppress activation of APCs and proliferation of Th1 and Th17 Cells as well as increase the expression of IL-10 *ex vivo*	^46^
Human BM-MSCs	Exosome 30-100 nm	Ultracentrifugation (100000 g 70 + 70 + 70 min)	TEM and western blotting	*Ex vivo* co-culture	Could induce the proliferation and migration of dermal fibroblasts derived from healthy donors and chronic wound patients, as well as induce angiogenesis of HUVECs *ex vivo*	^47^

EVs: extracellular vesicles; MVs: microvesicles; UCB-MSCs: umbilical cord blood-derived mesenchymal stem cells; BM-MSCs: bone marrow-derived mesenchymal stem cells; WJ-MSCs: Wharton’s jelly-derived mesenchymal stem cells; ESC-MSCs: embryonic stem cell-derived mesenchymal stem cells; HPLC: high performance liquid chromatography; TEM: transmission electron microscopy; SEM: scanning electron microscopy; NTA: nanoparticle tracking analysis; NOD/SCID mice: nonobese diabetic/severe combined immunodeficiency mice; Treg: regulatory T cells; Th: helper T cells; CX3CL1: C-X3-C motif chemokine ligand 1; HUVECs: human umbilical vein endothelial cells; AKI: acute kidney injury; TBI: traumatic brain injury; IDDM: insulin dependent diabetes mellitus; APCs: antigen presenting cells; EAU: experimental autoimmune uveoretinitis

MSCs have a supporting effect on *ex vivo* expansion of CD34^+^ HSCs.^60,61^ Early passages of MSCs, early stage of the co-culture, irradiated MSCs, and children’s BM-MSCs, further increase *ex vivo* expansion of HSCs in a more primitive fraction (CD34^+^/CD38-).^62-66^ Adding exogenous growth factors of HSCs in the co-culture medium with MSCs and forced expression of HSC-supportive factors by engineering in MSCs, further enhanced *ex vivo* expansion of HSCs.^67,68^ In this regard, CD34^+^ HSCs in the co-culture medium with MSCs as a feeder layer in the presence of exogenous cytokines such SCF, TPO, and FLT3L compared with cytokines and MSCs conditions alone, further enhanced their *ex vivo* expansion.^69,70^

MSCs in addition to the BM, also have been isolated from various tissues, including UCB, lung, dental pulp, fetal blood and liver, adipose tissue, skeletal muscle, cardiac tissues, placenta, synovial membrane, Wharton’s jelly, and amniotic fluid.^17,71-73^ MSCs from different sources induce the expansion of CD34^+^ HSCs in the co-culture system.^64,74-76^ However, BM-MSCs are significantly more efficient than other sources of MSCs such as Wharton’s jelly, amnion, and chorion for the *ex vivo* expansion of CD34^+^ HSCs.^76^ While in the another study it has been shown, Wharton’s jelly-derived mesenchymal stem cells (WJ-MSCs) have better potential to support *ex vivo* expansion of CD34^+^ HSCs in a more primitive condition than BM-MSCs.^77^ Moreover, no important difference observed with BM-MSCs compared to UCB-MSCs, and amniotic fluid.^76^ One group in 2015 reported that placenta-derived mesenchymal stem cells (P-MSCs) are better feeder layer for *ex vivo* expansion of HSCs than UCB-MSCs. They also reported, there is similar potential between BM-MSCs and P-MSCs for *ex vivo* expansion of HSCs.^64^ Additionally, it has been shown that adipose tissue-derived mesenchymal stem cells (AD-MSCs) with faster expansion rate than BM-MSCs, could support *ex vivo* expansion of CD34^+^ peripheral blood HSCs to the higher rate than BM-MSCs.^78^

Da Silva et al^79^ showed that direct cellular interactions between MSCs and CD34^+^ HSPCs, enhance *ex vivo* expansion of CD34^+^ and CD34^+^/CD38- HSPCs. Similar results from other studies also showed the importance of this result.^80,81^ Moreover, it has been shown that in the co-culture system of MSCs and HSCs, MSCs surface is suitable for the *ex vivo* expansion of HSCs.^14^ In contrast to these studies, a study demonstrated indirect contact of MSCs with HSCs compared to direct contact, further enhance proliferation and *ex vivo* expansion rate of HSCs.^82^

In the recent years, methods employed for the *ex vivo* expansion of HSCs, use natural or synthetic biomaterials in 2-dimensional (2D) or 3-dimensional (3D) culture systems. These biomaterials closely mimic the characteristics of *in vivo* HSC niche and so control the expansion and differentiation potential of HSCs. MSCs led to further expansion of CD34^+^ HSPCs in the 3D co-culture system compared to the 2D co-culture system.^83^ In the 3D collagen-based co-culture with MSCs, HSCs in contact with the collagenous matrix had higher expansion potential for a more primitive phenotype (CD34^+^, CD38-).^84^ MSCs in the 3D fibrin scaffold enhance further *ex vivo* expansion of CD34^+^ HSCs than collagen and poly-epsilon-caprolactone (PCL) scaffolds.^85^ It was also reported that 2D fibrin-based cultures without MSCs enhance further expansion of CD34^+^ HSCs than 2D PCL based cultures.^86^ Differentiated Osteoblasts from BM-MSCs have a supporting effect on *ex vivo* expansion of HSCs in the 2D co-culture system.^87^ In this regard, a group in 2016 reported that 3D co-culture system of BM-MSCs and differentiated osteoblasts from BM-MSCs with HSCs (3D mix) on human bio-derived bone scaffolds promote *ex vivo* expansion of CD34^+^ HSCs. On the other hand, *ex vivo* expansion of CD34^+^ HSCs in the 3D mix co-culture system significantly higher than 3D co-culture system of differentiated osteoblasts with CD34^+^ HSCs. Additionally, *ex vivo* expansion of CD34^+^ HSCs in the 3D MSCs with CD34^+^ HSCs co-culture system is close to 3D mix co-culture system.^88^ Huang et al^89^ in 2016, also used human bio-derived bone scaffolds to establish a 3D co-culture system for BM-MSCs and human umbilical vein endothelial cells (HUVECs) with HSCs, that led to long-term *ex vivo* expansion of HSCs. Meanwhile, *ex vivo* expansion of HSCs in the 3D mix co-culture system (BM-MSCs and HUVECs with HSCs) significantly higher than 3D BM-MSCs and 3D HUVECs. Regarding the above-mentioned studies developing the new and improved biomaterials are needed for 3D co-culture systems for improving HSCs *ex vivo* expansion. However, these efforts must answer the demands in the clinical setting, especially improve the HSCs engraftment.

### The effect of MSCs on HSCT and GVHD management following HSCT 

HSCT achieved clinical therapeutic progress in the recent decades and is a useful and practical solution for cell therapy of many malignant and non-malignant hematological disorders, and immune system disorders.^90^ The first transplantation of HSCs are successfully performed in HLA-matched siblings from a UCB of a sister in 1988, was used to treat her younger brother with severe Fanconi anemia.^91^ Several factors affect the HSCs successful engraftment after transplantation, including dose of transplanted stem cells, the intensity of the primary treatment regimen, the HLA compatibility level between donor and recipient, the T cells amount in the graft, and the immunosuppression intensity after transplantation.^92-94^ MSCs possess various properties that by using them could promote HSCT and help to prevent graft rejection and GVHD following HSCT. These properties including secreted various cytokines and growth factors, extracellular adhesion molecules, the ability to differentiate into different stromal cells, tendency to homing into damaged and inflamed tissues after infusion, the immunoregulatory and anti-inflammatory effects on different subsets of immune system cells, easy isolation, and *ex vivo* expansion.^95-100^

MSCs overexpressing C-X-C motif chemokine receptor 4 (CXCR4) that generated by transduction in co-transplantation with HSCs, promote HSCT and hematopoiesis in lethally irradiated mice.^100^ Another study showed that overexpression of CXCR4 on MSCs with a cocktail of cytokines, such as SCF, FLT3-L, IL-6, HGF (hepatocyte growth factor), and IL-3, accelerate the hematopoietic recovery after transplantation in nonobese diabetic/severe combined immunodeficiency (NOD/SCID) mice.^101^ Moreover, hematopoietic engraftment process enhanced by culture-expanded UCB-MSCs transfected with cytokine genes required for growth of HSCs such as G-CSF and SCF, that co-transplanted with UCB-HSCs into NOD/SCID mice.^102^ Genetically modified MSCs that express stromal cell-derived factor-1/homeobox B4 (SDF-1/HOXB4) fusion protein in co-transplantation with human UCB-HSCs enhance survival and hematopoietic recovery in irradiated mice after transplantation.^103^

MSCs from sources such as adipose tissue and UCB have a potential as BM-MSCs for enhancing the engraftment of HSCs into NOD/SCID mice. UCB-MSCs from different donors have different effects in enhancing the engraftment of HSCs.^104^ On the contrary, it has been shown that AD-MSCs compared to BM-MSCs through producing a higher level of C-X-C motif chemokine ligand 12 (CXCL12), further promote HSCs homing and engraftment in a mouse model.^105^ Van der Garde et al^77^ in 2015 showed that WJ-MSCs or BM-MSCs in co-transplantation with UCB-HSCs have similarly enhanced effects on hematopoietic recovery into NOD/SCID mice. Moreover, WJ-MSCs have better potential to support CD34^+^ HSCs in a more primitive condition than BM-MSCs. Since WJ of UCB is a waste product and WJ-MSCs have some advantages such as higher rate of expansion, obtained at low-cost, minimal ethical concerns, lower immunogenicity, painless isolation method with any risk or harm to the donor compared to BM-MSCs. Therefore, WJ-MSCs can be used as a practical alternative source for HSCT. HSPCs derived from umbilical cord blood, expanded on WJ-MSCs in HP01 serum‏-free medium without adding exogenous cytokines, improve the hematopoietic engraftment in NOD/SCID mice.^106^ Without adding exogenous cytokines, *ex vivo* expanded HSCs on MSCs improve long-term hematopoietic engraftment in NOD/SCID mice.^107^ While in some studies, it has been shown that *ex vivo* expanded HSCs on MSCs in the presence of cocktails of cytokines such as TPO, SCF, G-CSF, and FLT3-L, enhance short-term hematopoietic engraftment in NOD/SCID mice.^108,109^ A group showed that combination of *ex vivo* expanded CD34^+^ umbilical cord blood cells and unmanipulated CD34^+^ umbilical cord blood cells on MSCs improve engraftment and decrease the time of platelet and neutrophil recovery after HSCT in patients with hematological malignancies compared with unmanipulated CD34^+^ umbilical cord blood cells only.^110^ Co-transplantation of MSCs with CD34^+^ cord blood cells into NOD/SCID mice, improved hematopoietic engraftment and platelet recovery after transplantation, and *ex vivo* expanded CD34^+^ cord blood cells with TPO improved early platelet recovery after transplantation, while co-transplantation of TPO-expanded CD34^+^ cord blood cells and MSCs into NOD/SCID mice not only had no synergistic effect on these results but also caused the risk of very low engraftment or rejection.^111^

Co-transplantation of adipose tissue-derived insulin-secreting MSCs (AD-IS-MSCs) that generated *ex vivo* with BM-HSCs into insulin-dependent diabetes Mellitus (IDDM) patients is a safe and efficient treatment choice for IDDM, and autologous co-infusion show a supported effect of the decreased need to exogenous insulin and therefore better long-term control of hyperglycemia compared with allogeneic co-infusion.^112^ Researchers in 2015 reported that co-transplantation of mouse AD-MSCs with HSCs, improve HSCs engraftment in an autologous mouse model. As respects, MSCs enhance the HSCT in allogeneic or xenogeneic transplantation model and this is probably due to the immunomodulatory features of MSCs, and this study performed in an autologous transplantation model, so no immune system rejection expected in this model. Therefore, strongly suggested that supportive hematopoietic engraftment effect of autologous AD-MSCs not due to the paracrine effects, but is due to direct contact between the HSCs and the AD-MSCs.^113^ Co-transplantation of autologous MSCs with HSCs, not only promote the recovery of platelets and neutrophils but also promote early recovery of T cell subsets and T cell reconstitution (especially reconstitution of naive CD4^+^ T cells) in patients with malignant lymphomas.^114^ Also, it has been shown that co-transplantation of autologous HSCs and allogeneic MSCs enhance CD4^+^ CD25^+^ FoxP3^+^ regulatory T cells (Treg cells) after transplantation in a case report of a patient who suffered from refractory systemic lupus erythematosus (SLE).^115^

HSCs engraftment effect of AD-MSCs is dose-dependent, and high doses of AD-MSCs significantly increase HSCs engraftment after transplantation, in line with the studies in NOD/SCID mice that co-transplanted with human HSCs and human MSCs.^113,116,117^ On the other hand, higher doses of MSCs might decrease engraftment of HSCs.^117^ Moreover, co-transplantation of MSCs in a dose-dependent manner not only increase HSCs engraftment but also decrease GVHD.^118^

There are some complications such as GVHD, relapse, tissue damage due to the intensity of the treatment regimen, and infection that are dangerous for life in HSCT patients and they can restrict the widespread application and successfulness of HSCT therapy.^119^ GVHD is the most common complication that causes death after allogeneic HSCT and complicated inflammatory response that is known by the increased release of pro-inflammatory cytokines, activation of many types of donor T cells, and subsequently led to tissue damage in healthy tissues of the recipient.^119^ There are some prophylactic procedures that used for reducing the severity and incidence of GVHD after allogeneic HSCT such as *ex vivo* depletion of donor T cells by active depletion or enrichment of CD34^+^ HSCs and immunosuppressive agents.^120,121^ Nevertheless, *ex vivo* depletion of donor T cells causes an increased rate of graft versus leukemia (GVL) effect, infection because of the removing of donor T cells, and relapse. Moreover, immunosuppressive agents that are first-line factors for treatment of GVHD associated with low response rate, decreased immune system reconstitution in patients, and the increased risk of GVL effect and opportunistic infections.^120,121^ For these reasons, new strategies are needed to prevent and treat the GVHD.

MSCs due to their immunomodulatory, anti-inflammatory, and tissue repair features could be effective in the field of GVHD treatment and prevention.^119^ BM-MSCs reduce the incidence of GVHD in mice that received allogeneic UCB transplantation.^122^ While some works suggested that MSCs cannot prevent GVHD^123^ or MSCs can decrease the incidence of GVHD but cannot promote hematopoietic recovery after transplantation.^99^ Also, Han et al. reported that co-infusion of MSCs with haploidentical HSCs compared to infusion of HSCs alone can reduce the time of neutrophil recovery after engraftment but cannot reduce the incidence of GVHD and has no impact on the infection and relapse in HSCT patients.^124^

MSCs through an increased in the Treg cells content, decreased donor T cells infiltration into target organs, increased expression of cytotoxic T lymphocyte antigen 4 (CTLA-4) molecule that expresses on Treg cells, and decreased of CD80/86 expression on recipient splenic dendritic cells (DCs) could suppress or reduce the incidence and severity of GVHD.^54,125^ In this setting, a recent study showed that third-party BM-MSCs administration decrease cutaneous sclerodermatous GVHD by blocking of immune effector cells infiltration into skin and this effect as a result of reduced expression of chemokines in skin such as C-C motif chemokine ligand 1 (CCL1), CCL3, CCL8, CCL17, and CCL22 and chemokine receptors such as C-C motif chemokine receptor 8 (CCR8) and CCR4 on CD4^+^ T cells and CCR1 on CD11b^+^ monocytes and macrophages.^126^ Another study showed that repeated infusions of UCB-MSCs can decrease the incidence of chronic GVHD after HLA-Haploidentical HSCT through an increased number of memory B cells and Treg cells and a decreased number of natural killer cells (NKs), as well as the increased ratio of Th1 cells to Th2 cells.^127^ BM-MSCs combined with conventional immunosuppressive agents are effective to refractory steroid-resistant acute GVHD, and decrease the incidence and severity of chronic GVHD by repairing damaged thymus and inducing the production of CD4^+^ CD25^+^ Foxp3^+^ Treg cells, although no differences observed in the incidence of infection and tumor relapse between patients with or without MSCs treatment.^128^ Third party MSCs through the increased numbers of IL-10^+^ FoxP3^+^ Treg cells, decreased numbers of pro-inflammatory Th17 cells, shifted immune system responses toward Th2 cells, increased the ratio of CD4^+^ T cells to CD8^+^ T cells, and increased secretion of IL-2, improve the treatment of steroid-refractory acute GVHD.^129^ Whereas above-mentioned studies demonstrated that the number of Treg cells was lower in the onset of GVHD and MSCs through an increased number of Treg cells could decrease the incidence of GVHD, some studies demonstrated that the number of Treg cells in the onset of GVHD was higher or MSCs infusion cannot increase the number of Treg cells and these issues complicate the role of Treg cells in the reduction of GVHD.^130,131^ The relevant information about the recent clinical applications of MSCs infusion to treat GVHD is summarized in Table 2.

### The effect of MSC-EVs on HSCT and GVHD management following HSCT

EVs are the significant regulators in the determination of HSCs fate. In the investigation that performed by Ratajczak et al^142^ it has been shown that mouse embryonic stem cell-derived microvesicles (ESC-MVs) considerably improve the survival and *ex vivo* expansion of murine HSPCs. As well as, co-culture of MVs derived from megakaryocytes (Mks) with HSPCs induce differentiation of HSPCs toward mature Mks lineages without adding exogenous TPO.^143^ Mesenchymal stem cell-derived microvesicles (MSC-MVs) enhance the *ex vivo* expansion of cord blood CD34^+^ HSCs and cord blood-derived mononuclear cells (CB-MNCs). But this enhancement was lower compared with the enhancement by MSCs alone. This is because of, various of growth factors secreted by MSCs in addition to MVs. Moreover, MSC-MVs enhanced further expansion of HSCs, when added to the co-culture system of MSCs and HSCs.^12^ The MSC-MVs through Wnt/*β*-catenin signaling pathway increase *ex vivo* expansion, self-renewal, and block differentiation of HSCs. MSC-MVs contain miRNAs that have regulatory effects on HSCs and miRNAs that target genes that have inhibitory effects on Wnt/*β*-catenin signaling pathway.^12^ De Luca et al. demonstrated that MSC-EVs miRNAs and Piwi-interacting RNAs (piRNAs) inﬂuence HSCs gene expression pattern such as inducing cell survival, inhibiting apoptosis, and decreasing cellular differentiation to the all hematopoietic lineages. As well as, MSC-EVs miRNAs through an increased expression of CXCR4 in the CD34^+^ HSCs that transfected with MSC-EVs miRNAs or treated with MSC-EVs, cause increased migration of CD34^+^ HSCs from peripheral blood (PB) to BM niche, significantly increase HSCs engraftment in NOD/SCID/IL-2Rγnull (NSG) mice.^24^ On the contrary, another study showed that MSC-EVs treatment induces the decreased quiescence and expansion of HSCs and increased differentiation of HSCs to myeloid progenitors. MSC-EVs promoted HSCs differentiation via cell surface interaction with Toll-like receptor 4 (TLR4) instead of their cargo. This interaction led to activation of myeloid differentiation primary response (MyD88) adaptor protein and NF/κB transcription factor.^144^ MSC-EVs or whole BM cells derived EVs can decrease *ex vivo* radiation injury to murine HSCs cell line by stimulating of HSC proliferation, suppression of DNA destruction and apoptosis. But *in vivo* effect of MSC-EVs to decrease radiation injury to murine marrow HSCs was partial. Also, overexpression of miRNAs that are abundant in MVs in the murine HSCs cell line could partially decrease the radiation injury.^145^

**Table 2:** Clinical use of MSCs infusion for treatment of GVHD

**Clinical Context**	**Number of patients**	**MSCs source**	**The dose of MSCs**	**Source of transplanted HSCs**	**Outcome**	**Ref**
steroid-resistant grade II-IV acute and chronic GVHD after allogeneic HSCT	40 adults and children patients	Third party PL-expanded BM-MSCs	Median 1.5 x 10^6^ cells/kg Median 3 dose	BM, UCB, and PB. HLA-matched, HLA-mismatched, and Haploidentical	OR: 67.5%; CR: 27.5%; PR: 40.0%. No severe toxicity, better in grade II and children.	^132^
steroid-resistant grade III-IV acute GVHD after allogeneic HSCT	46 adults and children patients	Third party FBS-expanded BM-MSCs	Median 6.81 × 10^6^ cells/kg Median 3 dose	BM, UCB, and PB. HLA-matched and HLA-mismatched	OR: 50%; CR: 6.5%; PR: 30.5%; TPR: 13%. severe transient side effects during cell injection: 4.3%; No acute or late side effects.	^133^
steroid-resistant grade I-IV acute or chronic GVHD after allogeneic HSCT	11 children patients	Third party PL-expanded BM-MSCs	Median 1.2 × 10^6^ cells/kg 2 dose	BM, UCB, and PB. HLA-matched and HLA-mismatched	OR: 71.4%; CR: 23.8%; PR: 47.6%. No acute or late side effects, better in acute GVHD.	^134^
steroid-resistant grade III-IV acute GVHD after allogeneic HSCT	13 patients	Third party PL-expanded BM-MSCs	Median 0.9 × 10^6^ cells/kg Median 2 dose	PB. HLA-matched and HLA-mismatched	OR: 54%; CR:7.5%; PR: 7.5%; MR:39%. No toxicity during or quickly after the injection.	^135^
steroid-resistant grade III-IV acute GVHD after allogeneic HSCT	37 children patients	Third party FBS-expanded BM-MSCs	1–2 × 10^6^ cells/kg Median 2 dose	BM, UCB, and PB. HLA-matched, HLA-mismatched, and Haploidentical	OR: 86%; CR: 65%; PR: 21%. Better OS, if MSC treatment quickly after the beginning of acute GVHD.	^136^
steroid-resistant grade I-IV acute GVHD after allogeneic HSCT	58 patients	Third party PL-expanded BM-MSCs	Median 0.99 × 10^6^ cells/kg Median 2 dose	BM and PB. HLA-matched and HLA-mismatched	OR: 47%; CR: 9%; VGPR: 9; PR: 29%. No better OS compared to patients that have no MSCs infusion.	^137^
steroid-resistant chronic GVHD after allogeneic HSCT	23 patients	Third party BM-MSCs	1 × 10^6^ cells/kg 3 dose	BM and PB. HLA-matched and HLA-mismatched	OR: 87%. Increased number of CD5^+^ regulatory B cells.	^138^
steroid-resistant grade III-IV acute GVHD after allogeneic HSCT	25 adults and children patients	Third party BM-MSCs	2 × 10^6^ cells/kg 8 dose	BM, UCB, and PB. HLA-identical and HLA-mismatch	OR: 60%; CR: 24%; PR: 36%. No side effects.	^139^
Sclerodermatous chronic GVHD after allogeneic HSCT	4 patients	Third party BM-MSCs	1-2 × 10^7^ cells/kg 4-8 dose	BM. HLA-identical sibling	gradually improvement of the symptoms of chronic GVHD. Intra-bone marrow injection. No side effects. Increased ratio of Th1/Th2 cells.	^140^
steroid-resistant grade II-IV acute GVHD after allogeneic HSCT	25 patients	Third party PL-expanded BM-MSCs	Median 1.1 × 10^6^ cells/kg 2–4 dose	Not shown	OR: 71%; CR: 46%; PR: 25%. Lower toxicity.	^141^

GVHD: graft versus host disease; HSCT: hematopoietic stem cell transplantation; PL-expanded BM-MSCs: platelet lysate expanded bone marrow-derived mesenchymal stem cells; FBS-expanded BM-MSCs: fetal bovine serum expanded bone marrow-derived mesenchymal stem cells; BM: bone marrow; UCB: umbilical cord blood; PB: peripheral blood; OR: overall response; CR: complete response; VGPR: very good partial response; PR: partial response; TPR: transient partial response; MR: mixed response; OS: overall survival.

**Table 3:** Effects of MSCs-EVs on the HSCs expansion, HSCT, and GVHD

Source of EVs	Type of EVs and size	Isolation method	Identify method	Source of HSCs	Outcome	Ref
Human BM-MSCs	MVs 100-1000 nm	Centrifugation (16000 g 1 + 1 h)	Flowcytometry and TEM	Human cord blood CD34^+^cells	Enhance the *ex vivo* expansion of cord blood-derived CD34^+^cells	^12^
Human BM-MSCs	EVs 100-2000 nm	Ultracentrifugation (100000 g 70 + 70 min)	Flowcytometry and TEM	Human cord blood CD34^+^cells	Induce the *ex vivo* cell survival and decrease differentiation of cord blood CD34^+^ cells. Significantly increase *in vivo* HSCs engraftment in NSG mice	^24^
Mouse BM-MSCs and mouse AD-MSCs	EVs 100-400 nm	Ultracentrifugation (100000 g 2 h)	TEM, western blotting, and NTA	Mouse BM-HSPCs	Induce the decreased expansion of HSPCs and increased differentiation of HSPCs to myeloid progenitors *ex vivo*	^144^
Murine BM-MSCs and human BM-MSCs	EVs mean 249nm	Ultracentrifugation (100000 g 1 h)	TEM, western blotting, and NTA	Murine BM-HSCs	Decrease radiation injury to murine HSCs cell line *ex vivo*. Partially decrease radiation injury to murine marrow HSCs *in vivo*	^145^
	MVs mean 340nm	(10000 g 1 h)				
	Exosome mean 181nm	(100000 g 1 h)				
Human UCB-MSCs	EVs 30-100 nm	Ultracentrifugation (100000 g 2 + 2 h)	Flowcytometry and TEM	Mouse BM-HSCs	Decrease acute GVHD and increase the survival of recipient mice that undergo allogeneic HSCT. Show immunosuppressive effects *ex vivo*	^25^
Human BM-MSCs	Exosome Size not shown	Exosome isolation kit	Flowcytometry and electron microscopy	-	Attenuated GVHD in a mouse model via the exosome-associated adenosine signaling pathway in Th1 cells.	^131^
Human BM-MSCs	Exosome 99-123 nm (ZetaView®)	Ultracentrifugation (100000 g 2 h)	TEM, western blotting, and NTA	-	Decrease symptoms of the therapy-resistant acute GVHD patients. *in vivo* and *ex vivo* decrease of pro-inflammatory cytokines	^146^
	133-138 nm (NanoSight®)					

EVs: extracellular vesicles; MVs: microvesicles; UCB-MSCs: umbilical cord blood-derived mesenchymal stem cells; BM-MSCs: bone marrow-derived mesenchymal stem cells; AD-MSCs: adipose tissue-derived mesenchymal stem cells; TEM: transmission electron microscopy; NTA: nanoparticle tracking analysis; BM-HSPCs: bone marrow derived hematopoietic stem and progenitor cells; BM-HSCs: bone marrow derived hematopoietic stem cells; HPC: hematopoietic progenitor cells; GVHD: graft versus host disease; HSCT: hematopoietic stem cell transplantation.

MSC-EVs have immunomodulatory effects on the immune system response and therefore they can prevent GVHD, adjust active immune system responses or inflammation-associated disorders. In a study for the first time, it has been shown that MSC-derived exosomes infusion significantly improve symptoms of the steroid-resistant acute GVHD patients shortly after administration of exosomes without reported side effects as well as *in vivo* and *ex vivo* decrease of pro-inflammatory cytokines such as TNF-α, IL-1β, and IFN-γ has been observed.^146^ Additionally, Amarnath et al. identified that CD73 expressing exosomes derived from human BM-MSCs modulate GVHD in a mouse model through converting of ATP to adenosine, and this exosome-associated mechanism inhibits Th1 cells function in the mouse and therefore modulate GVHD.^131^ Finally, a group showed that MSC-EVs could decrease acute GVHD by modulating immune system responses in mice that undergo allogeneic HSCT, decrease the *in vivo* manifestations of acute GVHD, and therefore significantly increased the survival of recipient mice. They also suggested because of the safety concerns of MSCs clinical applications, MSC-EVs can use as an ideal alternative for preventing of acute GVHD after allogeneic HSCT.^25^ Recipient mice showed the important decrease of frequency and the absolute number of alloreactive T cells; an increased ratio of helper T cells to alloreactive T cells; decreased serum levels of pro-inflammatory cytokines such as TNF-α, IL-2, and IFN-γ; and increased serum levels of anti-inflammatory cytokines such as IL-10.^25^ Compatible with data from previous investigations.^44,147^

The therapeutic effect of MSC-EVs in the recent studies on HSCs expansion, HSCT, and GVHD is summarized in Table 3.

In addition to healthy tissues, MSC-EVs obtained from patient tissues could significantly control the determination of HSCs fate. In this regard, a group recently showed that MSC-MVs of myelodysplastic syndrome (MDS) patients have different cargo compared with MSC-EVs from healthy donors and could adjust CD34^+^ hematopoietic progenitor cells (HPCs) characteristics such as increasing cell viability and clonogenicity and changing of miRNA and gene expression pattern in the co-culture system.^148^ As well as, investigations on MSC-EVs of myeloproliferative neoplasm (MPN) patients, showed that their total miRNA content especially miRNA155 increased compared with healthy donors. MSC-EVs from both MPN patients and healthy donors could increase cell viability of CD34^+^ HPCs and MSC-EVs from MPN patients could increase the number of granulocyte-monocyte colony forming unit (CFU-GM) from neoplastic CD34^+^ HPCs in the co-culture system.^149^

## Conclusion

Many studies have been conducted on the co-culture and co-transplantation of MSCs with HSCs to improve HSCs expansion and the clinical potential of HSCT, prevention and treatment of GVHD, which emerging results showed different effects. During co-culture and co-transplantation with MSCs, HSCs undergo of either expansion or differentiation. Controlling the balance between expansion and differentiation of HSCs for HSCT and GVHD objectives is essential. For HSCT and GVHD objectives, it is essential to decrease differentiation and increase expansion and further studies are needed to answer this problem. However, there are some drawbacks about MSCs clinical applications such as increased incidence of pneumonia-related death in HSCT patients, the increased tumor progression, the uncontrolled differentiation of MSCs that led to ectopic tissue formation, pulmonary embolism due to intravenous infusion, and the undesired long-term side effects, Which challenged their clinical applications.^29,150,151^ Therefore, some recent studies used MSC-EVs because of the advantages that mentioned in the introduction, instead of MSCs for cell-based therapy in the field of HSCs expansion, engraftment, and GVHD. MSC-EVs in these studies showed that they can use as a practical alternative for improving HSCs expansion and engraftment, decreasing differentiation of HSCs and GVHD following HSCT. Their effects were lower than MSCs. These effects are probably due to the fact that MSCs exert their effects in the cell-based therapy by employing both intercellular direct contact and paracrine processes.

Whereas the well-defined molecular and cellular mechanisms that led to the supportive effect of MSC-EVs on hematopoiesis are still unknown, controversial, and probably are different from one condition to another. Moreover, at present, differential ultracentrifugation is the most widely used and gold standard method for isolation of MSC-EVs that performed through different protocols and therefore led to different results. For example, different populations of MSC-EVs obtained through different protocols of differential ultracentrifugation that they are different in bioactive cargoes.^145^ Additionally, MSCs heterogeneity, gender, and donor age may influence on MSC-EVs biological effects and cargoes because of these factors showed different effects on the functional features of MSCs.^152,153^ Therefore, further studies are needed to obtain comprehensive knowledge about MSC-EVs roles in the hematopoiesis and their isolation methods.

In the future, developing the combination of MSCs and MSC-EVs with factors that enhance self-renewal, block differentiation, and promote homing for HSCs engraftment purposes is essential. Moreover, we must have comprehensive knowledge about the changes that are occurring in the HSCs after inducing by MSC-EVs, the complete bioactive cargo that packaged into the MSC-EVs, the safety of MSC-EVs, and optimal dosage of MSC-EVs. Finally, methods for improving the storage, collection, purifying, and large-scale production of MSC-EVs that are cost-effective, less time-consuming and less labor-intensive should be developed and standardized.

## Acknowledgments

The authors are grateful to thanks, all research staff at the Umbilical Cord Stem Cell Research Center of Tabriz University of Medical Sciences, Tabriz, Iran for their generous support of this review.

## Ethical Issues

Not applicable.

## Conflict of Interest

The authors report no conflicts of interest in this review.
